# Complement Factor H-Related Proteins FHR1 and FHR5 Interact With Extracellular Matrix Ligands, Reduce Factor H Regulatory Activity and Enhance Complement Activation

**DOI:** 10.3389/fimmu.2022.845953

**Published:** 2022-03-22

**Authors:** Alexandra Papp, Krisztián Papp, Barbara Uzonyi, Marcell Cserhalmi, Ádám I. Csincsi, Zsóka Szabó, Zsófia Bánlaki, David Ermert, Zoltán Prohászka, Anna Erdei, Viviana P. Ferreira, Anna M. Blom, Mihály Józsi

**Affiliations:** ^1^ MTA-ELTE Complement Research Group, Eötvös Loránd Research Network (ELKH), Department of Immunology, ELTE Eötvös Loránd University, Budapest, Hungary; ^2^ MTA-ELTE Immunology Research Group, Eötvös Loránd Research Network (ELKH), Department of Immunology, ELTE Eötvös Loránd University, Budapest, Hungary; ^3^ Department of Translational Medicine, Lund University, Malmo, Sweden; ^4^ Department of Internal Medicine and Haematology, Semmelweis University, Budapest, Hungary; ^5^ Research Group for Immunology and Haematology, Semmelweis University-Eötvös Loránd Research Network (Office for Supported Research Groups), Budapest, Hungary; ^6^ Department of Immunology, ELTE Eötvös Loránd University, Budapest, Hungary; ^7^ Department of Medical Microbiology and Immunology, University of Toledo College of Medicine, Toledo, OH, United States

**Keywords:** factor H-related protein (FHR), extracellular matrix (ECM), complement regulation, factor H (FH), laminin, glomerular basement membrane (GBM), Bruch’s membrane (BM), kidney disease

## Abstract

Components of the extracellular matrix (ECM), when exposed to body fluids may promote local complement activation and inflammation. Pathologic complement activation at the glomerular basement membrane and at the Bruch’s membrane is implicated in renal and eye diseases, respectively. Binding of soluble complement inhibitors to the ECM, including factor H (FH), is important to prevent excessive complement activation. Since the FH-related (FHR) proteins FHR1 and FHR5 are also implicated in these diseases, our aim was to study whether these FHRs can also bind to ECM components and affect local FH activity and complement activation. Both FH and the FHRs showed variable binding to ECM components. We identified laminin, fibromodulin, osteoadherin and PRELP as ligands of FHR1 and FHR5, and found that FHR1 bound to these ECM components through its C-terminal complement control protein (CCP) domains 4-5, whereas FHR5 bound *via* its middle region, CCPs 3-7. Aggrecan, biglycan and decorin did not bind FH, FHR1 and FHR5. FHR5 also bound to immobilized C3b, a model of surface-deposited C3b, *via* CCPs 3-7. By contrast, soluble C3, C3(H_2_O), and the C3 fragments C3b, iC3b and C3d bound to CCPs 8-9 of FHR5. Properdin, which was previously described to bind *via* CCPs 1-2 to FHR5, did not bind in its physiologically occurring serum forms in our assays. FHR1 and FHR5 inhibited the binding of FH to the identified ECM proteins in a dose-dependent manner, which resulted in reduced FH cofactor activity. Moreover, both FHR1 and FHR5 enhanced alternative complement pathway activation on immobilized ECM proteins when exposed to human serum, resulting in the increased deposition of C3-fragments, factor B and C5b-9. Thus, our results identify novel ECM ligands of FH family proteins and indicate that FHR1 and FHR5 are competitive inhibitors of FH on ECM and, when bound to these ligands, they may enhance local complement activation and promote inflammation under pathological conditions.

## Introduction

The complement system, being a powerful effector arm of innate immunity, requires proper regulation to focus its activation on target cells, such as invading microbes and dying host cells, yet at the same time avoid unwanted damage to healthy host cells and tissues (1, 2). Dysregulation of the alternative pathway is associated with complement-mediated damage and is implicated in the pathomechanism of several diseases, including age-related macular degeneration (AMD), rheumatoid arthritis (RA) and the kidney diseases atypical hemolytic uremic syndrome (aHUS) and C3 glomerulopathy (C3G) (3, 4). Factor H (FH), a 155-kDa serum glycoprotein, is the major inhibitor of the complement alternative pathway and as such it inhibits complement activation at the level of the central complement component C3. FH binds to the C3b fragment of C3, and acts as a cofactor for factor I in the enzymatic inactivation of C3b; it also prevents assembly of the C3 convertase enzyme (C3bBb) of the alternative pathway and accelerates decay of this convertase once already formed, as well as regulates the C5 convertases (5, 6).

The human factor H protein family also includes the FH splice variant factor H-like protein 1 (FHL1) and five factor H-related (FHR) proteins. All FH family members exclusively consist of complement control protein (CCP) domains (also known as short consensus repeats). The FHRs show high but varying degree of amino acid sequence identity with the corresponding domains of FH, especially in their C-terminal part (3, 7–9). The FHR proteins lack domains related to the N-terminal CCPs 1-4 of FH, which are responsible for the FH complement inhibitory activity (8, 9). Due to the sequence similarity, FHRs share ligand binding properties with FH and they can all bind to C3b, suggesting a role for them in the modulation of complement activation; other ligands shared by some of the FHRs and FH include heparin, DNA and the pentraxins C-reactive protein (its monomeric or pentameric form) and pentraxin 3 (3, 7–11). However, their role in complement regulation has been controversial (8). For FHR5, a weak cofactor activity was reported at relatively high concentration compared to the physiological serum level of FHR5 (12). In surface convertase assays using purified proteins, FHR5 was shown to inhibit C5 conversion (13). FHR1 in turn was reported to inhibit complement at the C5 level and/or the terminal pathway (14), but this report was not confirmed in independent studies (10, 15–17). Recent studies showed that both FHR1 and FHR5 can compete with FH for binding to C3b, and to support assembly of the alternative pathway C3 convertase (C3bBb), thereby enhancing alternative pathway activation (7, 10). FHR5 also inhibits FH binding to DNA, pentraxins, malondialdehyde epitopes and to extracellular matrix (ECM) proteins (7, 11, 18). Thus, current evidence supports a role for FHR1 and FHR5 as competitive inhibitors of FH for binding to different ligands and instead of inhibiting complement as FH, FHR1 and FHR5 rather enhance complement activation (3, 7, 10, 11, 15, 19–21).

According to genetic studies, mutations in the *CFHR1* and *CFHR5* genes are associated with kidney and eye diseases where inappropriate or excessive complement activation is implicated at the glomerular basement membrane (GBM) or at the Bruch’s membrane (BM) (3, 8, 20–28). Quantitative and qualitative changes in FHR proteins apparently contribute to the pathological processes in diseases such as AMD, aHUS, C3G and IgA nephropathy (20, 21, 27, 28).

The GBM and BM are considered as specialized ECMs (29, 30). The ECM is mainly composed of collagen and/or elastin fibers, proteoglycans and glycoproteins. The GBM as a part of the glomerular filtration barrier mainly consists of collagen type IV, laminin and heparan sulphate among others (30). The multifunctional BM which is localized between the retinal pigment epithelium (RPE) and choroid, is mostly composed of elastin fibres, collagen fibres (mainly collagen type IV), laminin, fibronectin, heparan sulphate among other molecules (29).

Components of the ECM are not accessible to serum proteins under normal conditions. However, upon inflammation and tissue damage, ECM in the kidney and in the eye become exposed to interaction with complement components (31–34). Moreover, both anatomic sites are characterized by a fenestrated endothelium, and changes in the vasculature such as the loss of choriocapillaris that influences the Bruch’s membrane composition likely contribute to the pathogenic process (35). The ECM has no integral/inherent complement regulators such as membrane-bound complement inhibitors (e.g. CD55, CD59), therefore surface-bound FH provides the main protection from complement-mediated attack and damage (36, 37). It was demonstrated that FH binds to fibroblast- and endothelial cell-derived ECM (3, 38). Moreover FH binds to short leucine-rich repeat glycoproteins like fibromodulin, osteoadherin, and proline/arginine-rich end leucine-rich repeat protein (PRELP) (31, 32, 34, 39, 40), which are components of the ECM in the joints, kidney and eye (32, 33, 40). Next to FH, which keeps its regulatory activity when bound, both FHR1 and FHR5 bind to MaxGel, a fibroblast-derived ECM that is used as an *in vitro* model of ECM, and modulate complement activation (7, 10). Moreover, binding of FHR5 to human laminin has been described (18).

Based on the ligand binding similarities of FH and FHR proteins, we hypothesized that FHR1 and FHR5 can interact with various extracellular matrix components. The aim of the present study was to analyze FHR1 and FHR5 binding to selected extracellular matrix components and to determine how their binding affects the regulatory activity of FH and overall complement activation.

## Materials and Methods

### Proteins, Abs, and Sera

Recombinant human FHR1, FHR2, FHR3, FHR4 and FHR5 produced by Novoprotein (Shanghai, China) were purchased from Gentaur (Kampenhout, Belgium). Polyclonal goat anti-human FHR5 was purchased from R&D Systems (Wiesbaden, Germany). Purified human factor H (FH), C3, C3b, iC3b, C3c, C3d, factor I (FI), properdin (FP), goat anti-human FH and goat anti-human FB Ab were obtained from Merck (Budapest, Hungary). The anti-human FH mAb A254 and the anti-properdin mAb A235, and normal human serum (NHS) were from Quidel (obtained *via* Biomedica, Budapest, Hungary). Rabbit anti-human C3d, and HRP-conjugated goat anti-mouse Ig, rabbit anti-goat Ig and swine anti-rabbit Ig were obtained from Dako (Hamburg, Germany) and the HRP-labeled goat anti-human C3 Ab was from MP Biomedicals (Solon, OH). Human laminin, aggrecan, biglycan, decorin, collagen IV, fibronectin and vitronectin were purchased from Sigma-Aldrich (Budapest, Hungary).

The ECM proteins fibromodulin, osteoadherin, and PRELP were expressed recombinantly in HEK293 cells and purified using affinity chromatography due to the presence of His-tag (41).

Human properdin was purified as described in (42). Properdin oligomeric forms (dimers, P2; trimers, P3; and tetramers, P4) were isolated from pure properdin by size exclusion chromatography, as previously described (42). Briefly, pure properdin (5 mg) was loaded onto a Phenomenex BioSep-Sec-S4000 column (600 x 7.8 mm) with a guard column (75 x 7.8 mm) and eluted at a 0.5 ml/min flow rate in PBS. Purified, physiological forms of properdin were stored at 4°C and used within 2 week of separation (42, 43).

### Protein Expression and Purification

Recombinant human FHR5 fragments comprising CCPs 1-4, CCPs 3-7, and CCPs 8-9 were amplified by polymerase chain reaction using codon-optimized human FHR5 DNA template and specific primers (**Table 1**), and cloned into the pBSV-8His Baculo-virus expression vector (44). The proteins were expressed in *Spodoptera frugiperda* (Sf9) cells and purified by nickel-affinity chromatography. Purified proteins were analyzed by Western blot using 10% SDS-PAGE under non-reducing conditions and by silver staining.

**Table 1:** The primers used to generate the FHR5 fragments used in this study.

Primer name	Length (nt)	Optimal temperature (°C)	Sequence (5’- 3’)
**optFHR-5 CCP1-4 fw**	32	76.6	ATGTAACTGCAGGGCACTCTCTGTGATTTCCC
**optFHR-5 CCP1-4 rev**	25	77.2	ATATAACCCGGGGACGCATGTGGGC
**optFHR-5 CCP3-7 fw**	35	85.3	ATAATAGCGGCCGCAAAGGGCGAGTGTCACGTCCC
**optFHR-5 CCP3-7 rev**	37	86.5	ATAATACCCGGGTGCCACGCAACGTGGCAATGACTGC
**optFHR-5 CCP8-9 fw**	32	76.6	AGATATCTGCAGGAGTCGACCGCTTACTGTGG
**optFHR-5 CCP8-9 rev**	31	77.5	AGTAATCCCGGGCTCACAGATTGGGTACTCG

### Protein Microarray

ECM proteins (0.5 mg/ml), gelatin (0.5 mg/ml) and MaxGel (0.8 mg/ml) were printed onto nitrocellulose-covered slides in triplicates. Air-dried slides were washed three times with PBS containing 0.05% Tween 20 and blocked with 4% BSA. In binding assays, the immobilized ECM proteins were incubated with FHR1 (5 µg/ml; 25 µg/ml; 50 µg/ml) or FHR5 (0.5 µg/ml; 5 µg/ml; 20 µg/ml), then detected with goat anti-human FH or goat anti-human FHR5 followed by Alexa-647 labeled anti-goat IgG.

To measure competition, printed proteins were incubated with 25 µg/ml FH with or without FHR1 (5 µg/ml; 25 µg/ml; 50 µg/ml) or FHR5 (0.5 µg/ml; 5 µg/ml; 20 µg/ml). Bound FH was detected using monoclonal mouse anti-FH Ab (A254) that does not cross-react with FHR1 and FHR5, and Alexa-546-conjugated goat anti-mouse IgG. After scanning, fluorescence intensities were calculated as median of the triplicates and background was subtracted.

Complement activation on protein microarray was analyzed by incubating immobilized ECM components with 20% NHS in the presence or absence of recombinant FHR1 (50 µg/ml) or FHR5 (20 µg/ml) diluted in DPBS containing Mg^2+^ and Ca^2+^ (Lonza). C3 deposition was detected with Alexa-555 labelled anti-C3 F(ab’)_2,_ and the sC5b-9 was detected with biotinylated monoclonal anti-sC5b-9 Ab and streptavidin-Alexa-488.

### Microtiter Plate Binding Assays

To analyze the binding of C3b, iC3b, C3c and C3d to FHR5 fragments, 5 µg/ml FHR5, FHR5 CCPs 1-4, CCPs 3-7, CCPs 8-9, and human serum albumin (HSA) as a negative control were immobilized on microtiter plate. After blocking with 4% BSA, 20 µg/ml C3b, iC3b, C3c and 10 µg/ml C3d were added to the corresponding wells. Bound proteins were detected with HRP-conjugated anti-human C3 or polyclonal rabbit anti-human C3d followed by HRP-conjugated swine anti-rabbit Ig. TMB High Sensitivity substrate solution (BioLegend) was used to visualize binding, and the absorbance was measured at 450 nm.

To measure binding in a reverse setting, 20 µg/ml C3b was immobilized and incubated with 20 µg/ml FHR5, the FHR5 fragments CCPs 1-4, CCPs 3-7 and CCPs 8-9 diluted in DPBS containing Mg^2+^ and Ca^2+^ (Lonza). HSA was used as a negative control protein. Bound proteins were detected with polyclonal goat anti-FHR5 Ab and HRP-conjugated rabbit anti-goat Ig antibody.

To further analyze the binding of C3 and C3(H_2_O) to FHR5, 10 µg/ml FHR5, CCPs 1-4, CCPs 3-7, CCPs 8-9 and as a negative control alpha-1-antitrypsin were immobilized in microplate wells, and incubated with 5 µg/ml C3, C3(H_2_O), and C3b, which was used as a positive control. C3(H_2_O) form was generated from C3 with ten freeze/thaw cycles. After washing steps, bound proteins were detected with HRP-conjugated anti-human C3 antibody.

To measure properdin binding, FHR proteins and C3b were immobilized at 5 µg/ml in DPBS containing Mg^2+^ and Ca^2+^ at 4°C overnight. After washing with DPBS containing 0.05% Tween-20, free binding sites were blocked by incubation with 4% BSA dissolved in DPBS containing 0.05% Tween-20 at 20°C for 1 hr, then 20 µg/ml of the various properdin forms diluted in DPBS containing Mg^2+^ and Ca^2+^ was added at 20°C for 1 hr. After washing, properdin binding was detected using the anti-properdin mAb A235 (1:1000) and the corresponding secondary Ab (1:1000).

To analyze FHR1 binding to ECM proteins, 5 µg/ml laminin, fibromodulin, osteoadherin, PRELP and HSA were coated and incubated with FHR1 (5 µg/ml or 10 µg/ml). Bound FHR1 was detected with goat anti-human FH and the corresponding secondary Ab. Inhibition of FHR1 binding to ECM proteins was measured by incubating the immobilized ECM proteins with 5 µg/ml FHR1 and simultaneously added mAb C18 (10 µg/ml), which recognizes CCP5 of FHR1, or mAb A255 (10 µg/ml), which does not recognize FHR1 and was used as a control. Binding of FHR1 was detected as described above.

For FHR5, ECM proteins were immobilized (10 µg/ml) and incubated with FHR5 and its fragments (CCPs 1-4, CCPs 3-7, CCPs 8-9) in equimolar concentrations (100 nM or 200 nM). After washing, bound proteins were detected as described above.

To measure the competition between FHR5 and FH for the binding to ECM proteins in microplate format, immobilized ECM proteins (10 µg/ml) were incubated with 50 µg/ml FH in the presence or absence of 20 µg/ml FHR5. HSA was used as a negative control. Bound FH was detected with monoclonal anti-human FH (A254) and HRP-conjugated goat anti-mouse Ig antibody.

### Complement Activation Assays

To measure complement activation and C3 convertase formation on ECM-bound FHR1/FHR5, microplate wells were coated with the ECM proteins (5 µg/ml or 10 µg/ml) and, after blocking with 4% BSA, incubated with 10% NHS diluted in 5 mM Mg^2+^-EGTA in the absence or presence of FHR5 (10 µg/ml) or FHR1(10 µg/ml; 20 µg/ml) for 30 min at 37°C. Deposition of C3 fragments and factor B (FB) was detected using HRP-conjugated anti-human C3, and goat anti-human FB followed by HRP-conjugated rabbit anti-goat Ig, respectively.

### Cofactor Assays

To assess the functional consequence of competition between FHR5 and FH, laminin was immobilized at 10 µg/ml and, after blocking with 4% BSA, 100 µg/ml FH was added in the absence or presence of 20 µg/ml FHR5. After washing, the wells were incubated with 140 nM C3b and 300 nM FI diluted in DPBS containing Mg^2+^ and Ca^2+^ for 1 hour at 37°C. Supernatants were collected and subjected to 7.5% SDS-PAGE and Western blotting under reducing conditions. C3 fragments were detected with a HRP-conjugated anti-human C3 Ab and an ECL detection kit (Merck).

### Statistical Analysis

Statistical analysis was performed using GraphPad Prism version 5.00 for Windows (GraphPad Software, San Diego, California). A *p* value <0.05 was considered statistically significant.

## Results

### FHR1 and FHR5 Bind to Several ECM Proteins

**Figure 1:**
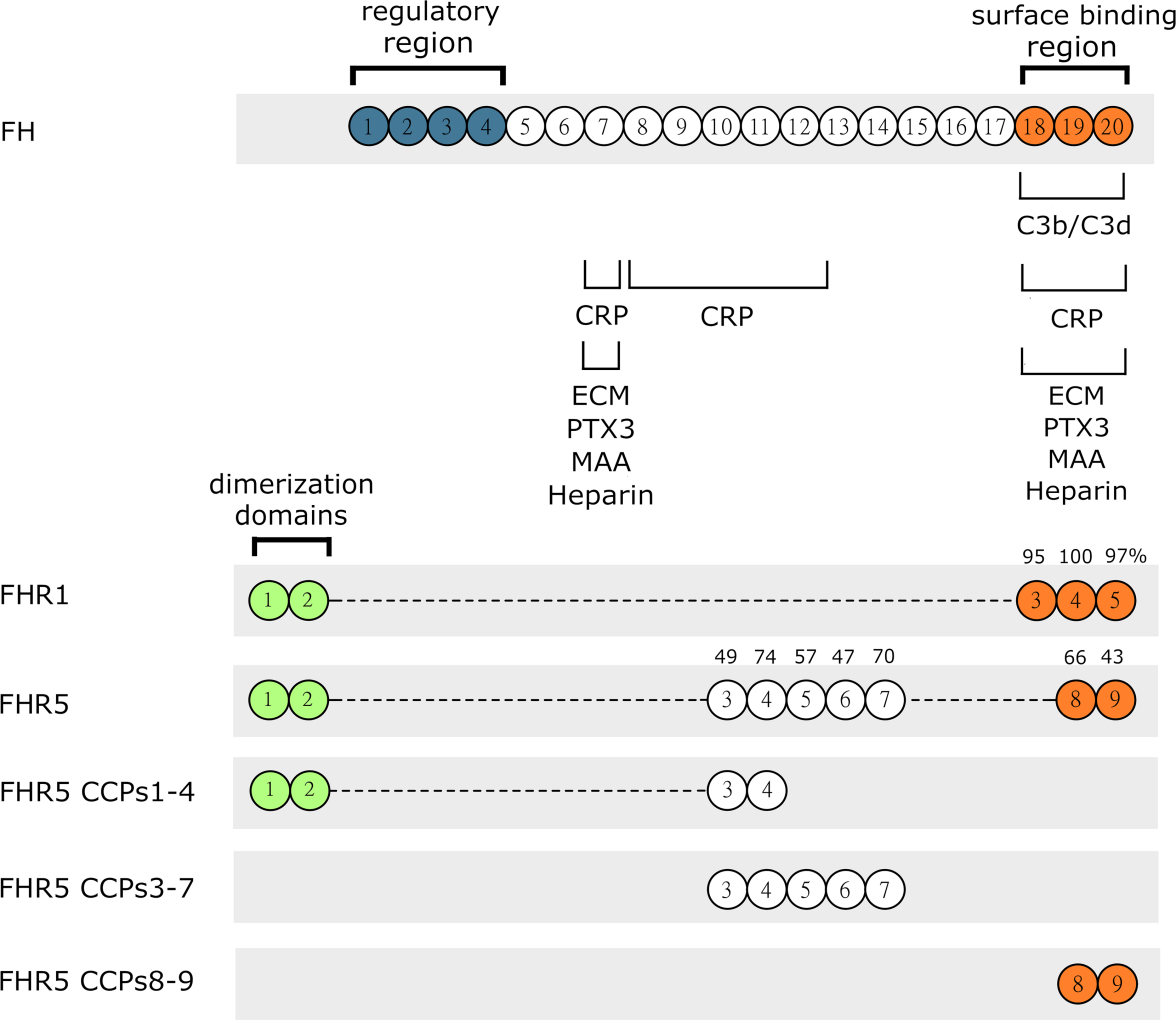
Schematic drawing of FH, FHR1, FHR5 and the recombinant FHR5 fragments used in this study. FH, FHR1 and FHR5 are composed of individually folding globular domains called complement control protein domains (CCPs) or also known as short consensus repeats (SCRs). CCPs 1-4 of FH mediate complement regulatory activity while the CCPs 7 and 18-20 are responsible for binding different ligands and surface recognition. The CCPs of FHR1 and FHR5 share high amino acid sequence identity with the corresponding domains of FH, indicated by numbers above. The C-terminal part is well conserved indicating similar or identical ligand and surface binding between FHR1, FHR5 and FH. FHR proteins lack the N-terminal regulatory domains CCPs 1-4 of FH. CCPs 1-2 of FHR1 and FHR5 are very similar to each other and responsible for formation of homo and heterodimers.

FH was previously shown to bind to several components of the extracellular matrix (ECM); moreover, FH represents the main alternative complement pathway inhibitor on ECM (31, 32), although its splice variant, factor H-like protein 1 (FHL-1) was reported as the major regulator in Bruch’s membrane (45). We hypothesized that, due to their sequence similarities with FH (**Figure 1**), FHR1 and FHR5 could interact with certain ECM components, which is also supported by recent data (7, 10, 18).

**Figure 2:**
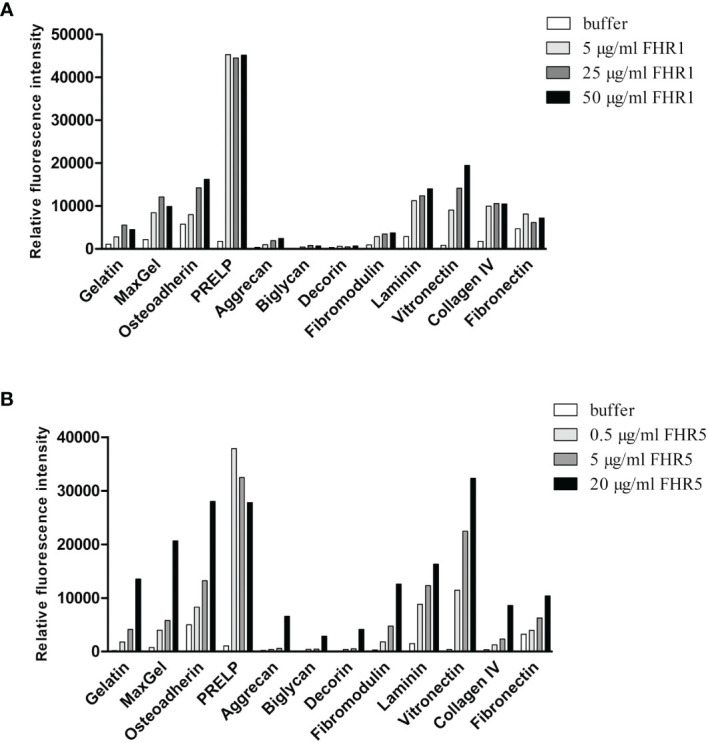
Binding of FHR1 and FHR5 to ECM components. FHR1 **(A)** and FHR5 **(B)** binding to ECM ligands was analyzed by protein microarray. ECM proteins, gelatin and MaxGel were printed onto nitrocellulose-covered slides in triplicates. Air-dried slides were washed and blocked with 4% BSA, then incubated with FHR1 or FHR5 in increasing concentrations. Bound proteins were detected with polyclonal goat anti-human FH or polyclonal goat anti-human FHR5 Ab and Alexa-647 labeled goat-IgG. A signal higher than the one obtained for the negative control protein gelatin was defined as the threshold for binding. Data are representative of two experiments.

**Figure 3:**
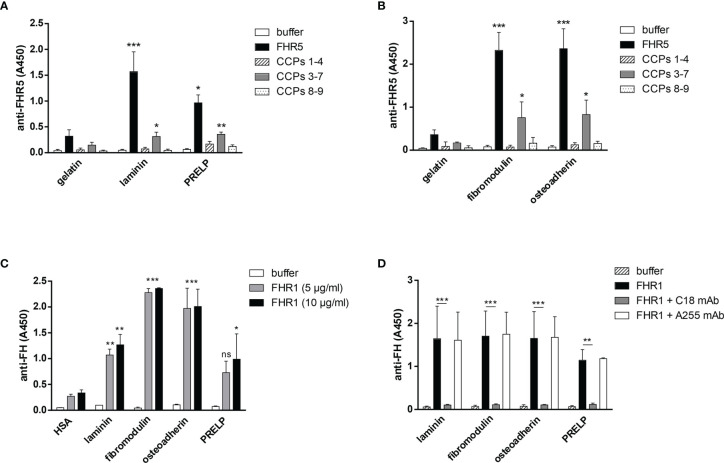
FHR5 binds through its middle part, CCPs 3-7, and FHR1 binds *via* its C-terminal domain to ECM proteins. In ELISA 10 µg/ml of gelatin, laminin, PRELP **(A)**, osteoadherin and fibromodulin **(B)** were immobilized in microplate wells and after blocking, incubated with 200 nM **(A)** or 100 nM **(B)** FHR5, CCPs 1-4, CCPs 3-7 and CCPs 8-9. Binding of FHR5 and its fragments was measured with goat anti-human FHR5 Ab. Data are means ± SD derived from three experiments. *p < 0.05, **p < 0.01, ***p < 0.001 one-way ANOVA, compared to the negative control, gelatin. **(C)** Laminin, fibromodulin, osteoadherin and PRELP were immobilized (10 µg/ml) in microplate wells. After blocking, FHR1 was added in increasing concentrations. Binding of FHR1 was detected with polyclonal goat anti-human FH and the corresponding secondary antibody. Data are means ± SD derived from three independent experiments. *p <0.05, **p <0.01, ***p <0.001 two-way ANOVA, compared to the negative control, HSA; ns, not significant. **(D)** Immobilized ECM proteins were incubated with FHR1 (5 µg/ml) in the presence or absence of the monoclonal Ab C18, which recognizes the CCP 5 domain of FHR1. We used monoclonal Ab A255 as a negative control. Bound FHR1 was detected as described earlier. Data are means ± SD derived from three independent experiments. *p < 0.05, **p < 0.01, ***p < 0.001 two-way ANOVA.

To test this hypothesis, first we used protein microarray technique. Various ECM proteins were printed onto nitrocellulose-covered slides in triplicates. The slides were incubated with increasing concentrations of FHR1 (**Figure 2A**) and FHR5 (**Figure 2B**), and their binding was detected using polyclonal antibodies to FH and FHR5, respectively. Both FHRs bound to several ECM proteins, including laminin, osteoadherin, PRELP and vitronectin, in a dose-dependent manner. Binding of FHR1 and FHR5 to laminin, osteoadherin and PRELP was confirmed by ELISA; in addition, prominent binding to fibromodulin was also detected in ELISA (**Figure 3**). This difference between the two types of assays might be due to the different surfaces and measurement methods.

To determine the binding sites for the analyzed ECM proteins within FHR5, recombinant FHR5 fragments comprising CCPs 1-4, CCPs 3-7, and CCPs 8-9 were generated, expressed in insect cells and purified (**Figure 1**, **Table 1** and **Supplementary Figure 1**). Binding of these FHR5 fragments to the immobilized ECM proteins was determined by ELISA using polyclonal anti-FHR5. We found that CCPs 3-7 of FHR5 bound to laminin, fibromodulin, osteoadherin and PRELP, indicating that FHR5 binds to these ECM proteins through its middle part, whereas the other tested FHR5 fragments did not bind (**Figures 3A, B**). FHR1 also bound to immobilized laminin, fibromodulin, osteoadherin and PRELP in ELISA (**Figure 3C**). Presumably, FHR1 binds through its most C-terminal domain to these ECM proteins, in contrast to FHR5, since when FHR1 was preincubated with the monoclonal antibody C18, which recognizes the CCP5 domain of FHR1, FHR1 binding to laminin, fibromodulin, osteoadherin and PRELP was inhibited. In contrast, the mAb A255, which binds to FH but not to FHR1, did not affect FHR1 binding in this assay (**Figure 3D**). Since laminin binding has not been described for FH before, we tested laminin binding to the FH fragments comprising the N-terminal regulatory domains CCPs 1-4, the middle part CCPs 8-14 (which contains domains related to FHR5) and the C-terminal CCPs 15-20 that contains domains homologous to the FHR1 C-terminus. Laminin bound only to CCPs 15-20, supporting a C-terminal binding site in both FH and FHR1 (**Supplementary Figure 2**).

### FHR1 and FHR5 Compete With FH for Binding to ECM Proteins

**Figure 4:**
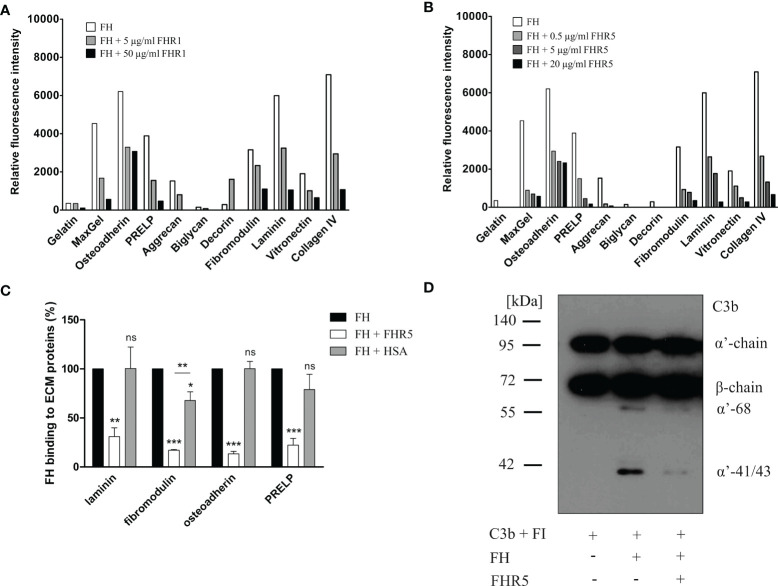
FHR1 and FHR5 compete with FH for binding to several ECM components, and FHR5 competitively inhibits the cofactor activity of FH. ECM proteins were printed on slides and incubated with 25 µg/ml FH together with increasing concentrations of FHR1 **(A)** or FHR5 **(B)**. Binding of FH was measured by monoclonal mouse anti-FH (A254) that does not recognize FHR1 or FHR5 and Alexa546-conjugated goat anti-mouse IgG. Data are representative of two experiments. **(C)** Competition between FHR5 and FH was also measured in ELISA. Laminin, fibromodulin, osteoadherin and PRELP (10 µg/ml of each) were immobilized and incubated with 50 µg/ml FH with or without 20 µg/ml FHR5. FH binding to ECM proteins was detected as described earlier. The values were normalized for FH binding (100%) and show means ± SD derived from three independent experiments. *p <0.05, **p <0.01, ***p <0.001, one-way ANOVA; ns, not significant. **(D)** Laminin (10 µg/ml) was immobilized and after blocking, the wells were incubated with 100 µg/ml FH with or without 20 µg/ml FHR5. Then, 140 nM C3b and 220 nM FI were added to the wells for one hour at 37°C. After incubation, supernatants were analyzed on 7.5% SDS-PAGE and Western blot. The blot was developed by using HRP-conjugated anti-human C3 Ab that recognizes C3b and its fragments except for C3d. The molecular mass marker is indicated on the left, and the C3b chains and the C3b α’-chain cleavage fragments are indicated on the right. Results are representative of three experiments.

To further analyze the interaction of FHR1 and FHR5 with ECM proteins and their effect on the functional activity of FH, we tested whether FHR1 and FHR5 compete with FH for binding to ECM proteins. In the protein microarray setup, the immobilized ECM proteins were incubated with FH together with increasing amounts of FHR1 and FHR5. Binding of FH was detected with an anti-FH mAb that does not recognize FHR1 and FHR5. Both FHR1 and FHR5 inhibited the binding of FH to osteoadherin, PRELP, fibromodulin, laminin, vitronectin and collagen IV in a dose-dependent manner, as well as to MaxGel that was used as a control (**Figures 4A, B**). Competition between FHR5 and FH was confirmed by ELISA where laminin, osteoadherin, fibromodulin and PRELP were immobilized in microplate wells and incubated with 50 µg/ml FH in the absence or presence of 20 µg/ml FHR5. FHR5 strongly inhibited FH binding to these ECM proteins (**Figure 4C**).

To study whether the competitive inhibition of FH binding by FHR5 impairs the complement regulatory activity by removing FH, a surface cofactor assay was performed. Laminin was immobilized in microplate wells and incubated with FH together with or without FHR5. After removing the unbound proteins by extensive washing, FI and C3b were added to the wells to allow for cleavage of C3b. After incubation, the supernatants were collected, the proteins were separated by SDS-PAGE and the C3b cleavage products were visualized by Western blot. FHR5 competitively inhibited the cofactor activity of FH on laminin coated surfaces as less C3b α’-chain was cleaved in the presence of FHR5 (**Figure 4D**).

### FHR5 Binds iC3b and C3d *via* Its C-Terminal Domains but It Does Not Bind C3c, and Also Interacts With C3 and C3(H_2_O)

**Figure 5:**
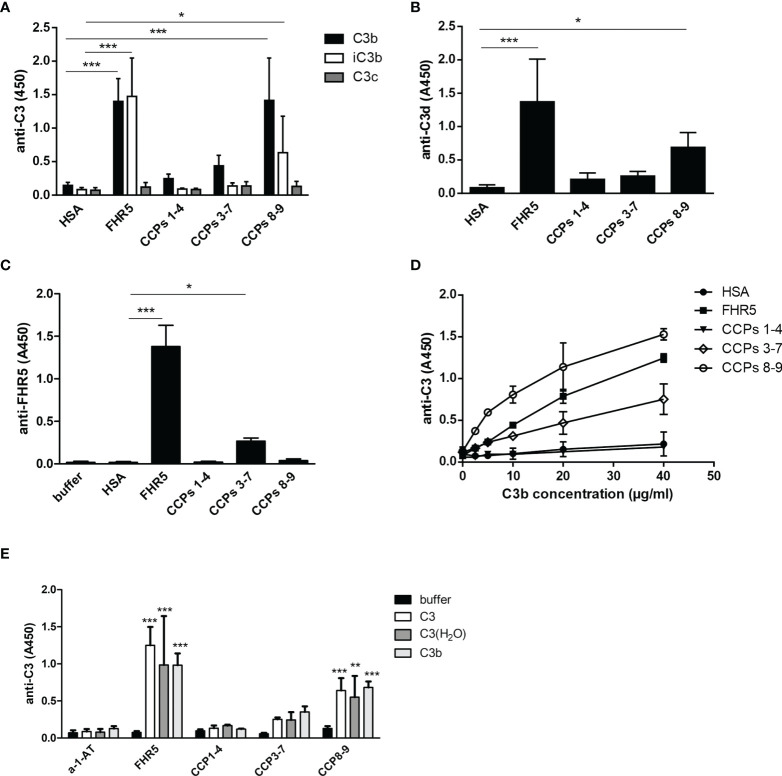
Binding of FHR5 and its fragments to C3b, iC3b, C3c and C3d. **(A)** FHR5, CCPs 1-4, CCPs 3-7, CCPs 8-9 and HSA were immobilized at 5 µg/ml in microplate wells. After blocking, 20 µg/ml C3b, iC3b and C3c were added. Bound proteins were detected using HRP-conjugated anti-human C3 Ab. **(B)** C3d binding to FHR5 also was measured by ELISA. FHR5 and its fragments were coated and incubated with 10 µg/ml C3d. Binding of C3d was measured with an anti-human C3d Ab and the corresponding secondary Ab. **(C)** In reverse setting, C3b was coated (20 µg/ml) and, after blocking, microplate wells were incubated with 20 µg/ml FHR5, CCPs 1-4, CCPs 3-7 and CCPs 8-9. HSA was used as a control protein. Binding of FHR5 and its fragments was detected by a polyclonal goat anti-FHR5 and HRP-conjugated secondary Ab. **(D)** Dose-dependent binding of C3b to FHR5 and its fragments was measured as shown in **(A)**; C3b was added in increasing concentrations. Data are means ± SD derived from five **(A–C)** or two **(D)** independent experiments. *p < 0.05, ***p < 0.001, one-way ANOVA. **(E)** Binding of C3, C3(H_2_O) and C3b to immobilized FHR5 and its fragments was measured by ELISA using HRP-conjugated polyclonal anti-C3 antibody. Data are means + SD derived from four experiments. **p < 0.01, ***p < 0.001, one-way ANOVA, compared to the negative control protein alpha-1-antitrypsin (a-1-AT).

All FHR proteins bind C3b, the main ligand of FH (3). FH has multiple recognition sites for C3b and other C3 fragments, but the C-terminal domains harbor the major binding site for surface-bound C3b. Because of the conserved FH C-terminal domains, FHRs are supposed to share this C-terminal C3b recognition site. We set out to determine the binding sites of various C3 fragments in the FHR5 protein, using recombinant FHR5 fragments comprising CCPs 1-4, CCPs 3-7 and CCPs 8-9 that cover the whole protein. FHR5 and its fragments were immobilized in microtiter plate wells and incubated with C3b, iC3b, C3c and C3d. FHR5 bound soluble C3b mainly *via* the C-terminal CCPs 8-9 (**Figures 5A, D**), in agreement with recent data (18). Similarly, both iC3b and C3d bound to the CCPs 8-9 fragment of FHR5, whereas C3c did not bind to any of the FHR5 fragments nor to the whole protein (**Figure 5A, B**). In reverse setting, where C3b was immobilized in microtiter plate wells and incubated with FHR5 fragments, CCPs 3-7 showed significant binding to surface-bound C3b (**Figure 5C**). Thus, FHR5 appears to have two C3b binding sites, one for surface-bound C3b and another for fluid-phase C3b, suggesting that FHR5 when bound to a surface either *via* deposited C3b or to other ligands exposed on the surface, such as bound pentraxins (11) or ECM proteins, it can potentially recruit C3b from fluid-phase and allow for alternative pathway activation by recruiting an active C3bBb convertase (7).

Since C3 fragments are generated upon complement activation, we also tested whether intact C3 and C3(H_2_O), which is physiologically constantly generated in serum at a low rate, can bind to FHR5. Purified C3 was subjected to freeze-thaw cycles to generate C3(H_2_O) (**Supplementary Figure 3**). Both C3 and C3(H_2_O) bound to the C-terminal fragment CCPs 8-9 of FHR5 (**Figure 5E**); however, it cannot be excluded that during the experiment C3 was ticking over and actually C3(H_2_O) what was bound.

### FHR1 and FHR5 Do Not Bind Properdin Directly

**Figure 6:**
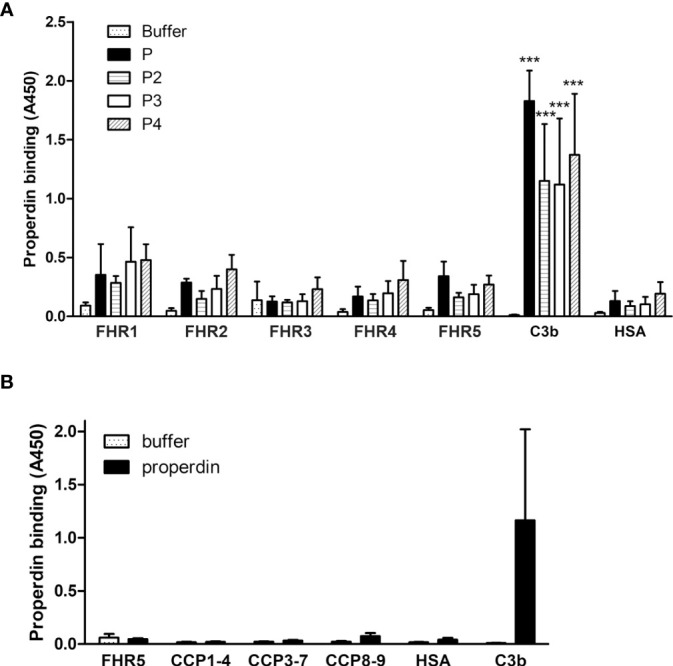
Analysis of properdin binding to FHRs. **(A)** The five FHR proteins, C3b as positive control protein and HSA as negative control protein were immobilized and binding of purified properdin (P) and the isolated properdin dimers, trimers and tetramers (P2, P3 and P4, respectively) was measured by ELISA using anti-properdin antibody. **(B)** Properdin binding to the FHR5 deletion mutants CCPs 1-4, 3-7 and 8-9 was measured as in **(A)**. Data are means + SD from three experiments. ***p <0.001, one-way ANOVA, compared to the negative control protein HSA.

Previously, we showed that FHR5 can activate the alternative pathway by binding C3b and recruiting an active C3 convertase enzyme, as showed by the deposition of C3b, Bb and properdin on immobilized FHR5. However, FHR5 did not bind FB or properdin directly (7). Rudnick et al. on the other hand described direct binding of properdin to FHR5 and suggested that properdin when bound to FHR5 recruits C3b and thus initiates complement activation (18). To resolve this contradiction, and since properdin notoriously tends to aggregate and generate higher order oligomers when stored for longer time or freeze-thawed (43), we analyzed the binding of purified commercial properdin (P), as well as purified properdin dimers, trimers, and tetramers (P2, P3, and P4, respectively). There was no significant binding of the physiologically occurring properdin forms to FHR5, nor to the other FHRs tested in parallel; however, there was prominent binding to C3b, used as a positive control (**Figure 6A**). In addition, since FHR5 CCPs 1-2 were proposed to harbor a properdin binding site (19), we analyzed our recombinant FHR5 fragments and no properdin binding to any of these was found in ELISA (**Figure 6B**). Altogether, these results suggest that physiological serum properdin forms are unlikely to bind directly to FHR5 and other FHRs.

### FHR1 and FHR5 Enhance Complement Activation on ECM Proteins Through the Alternative Pathway

**Figure 7:**
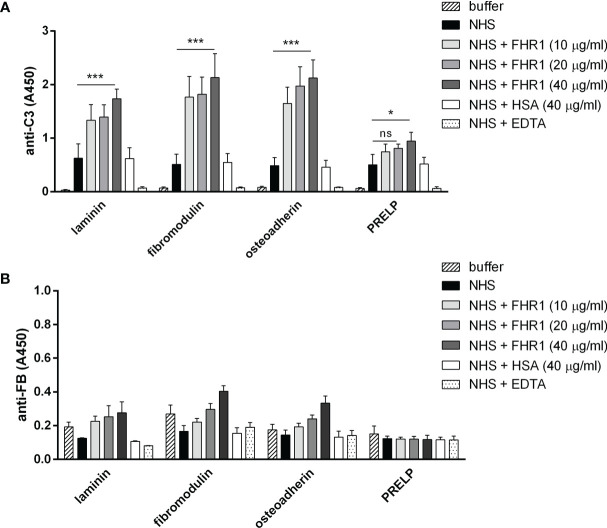
FHR1 increases C3 fragment deposition on ECM proteins. Immobilized ECM proteins were incubated with 10% NHS with or without FHR1 added in increasing concentrations (10 µg/ml, 20 µg/ml, 40 µg/ml) diluted in 5 mM Mg^2+^-EGTA. C3 fragment deposition **(A)** was measured with HRP-conjugated polyclonal anti-human C3 Ab and FB binding **(B)** was measured by goat anti-human FB and the corresponding secondary Ab. Data are means ± SD derived from four independent experiments. *p <0.05, ***p < 0.001, two-way ANOVA; ns, not significant.

**Figure 8:**
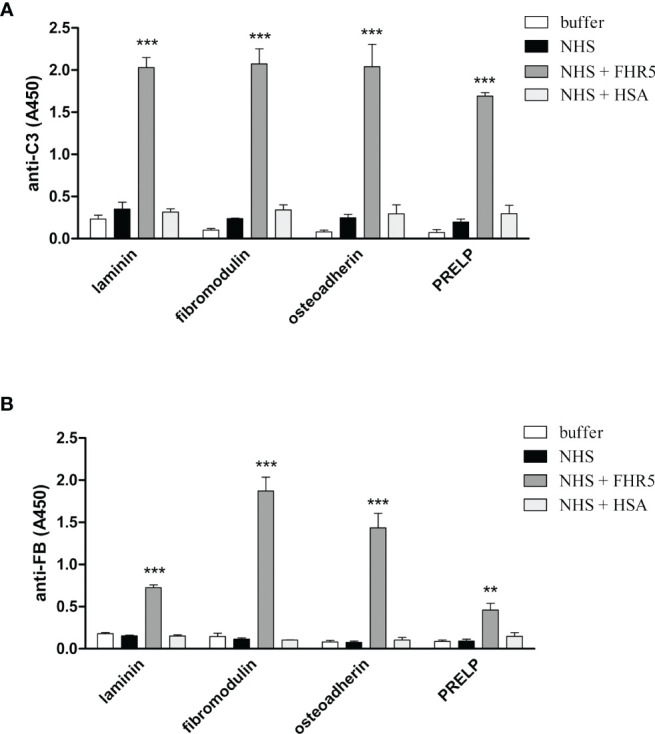
FHR5 causes enhanced C3 fragment deposition and supports alternative pathway activation when bound to ECM proteins. Immobilized ECM proteins were incubated with 10% NHS in the presence or absence of 10 µg/ml FHR5, supplemented with 5 mM Mg^2+^-EGTA which allows only alternative pathway activation. Binding of C3 fragments and factor B was detected with HRP-conjugated anti-human C3 Ab **(A)** and goat anti-human FB Ab **(B)**. Data are means ± SD derived from three experiments. **p < 0.01, ***p < 0.001, one-way ANOVA.

Competition between FH and FHR1 or FHR5 for binding to ECM is only one way how the regulation of complement activation can be compromised at such a surface. Previously, we demonstrated that both FHR1 and FHR5 allow formation of the alternative pathway C3 convertase when immobilized on microplate wells and rather support than inhibit complement activation. We tested whether FHR1 and FHR5 show this activity when bound to ECM proteins. To this end, ECM proteins were immobilized and incubated with NHS in the presence or absence of recombinant FHR1 or FHR5 in a buffer supplemented with 5 mM Mg^2+^-EGTA to allow only the activation of alternative pathway. Generation of the alternative pathway C3 convertase on the surface bound FHR proteins was confirmed by measuring deposition of C3b and factor B (FB). Both FHR1 and FHR5 significantly enhanced the amount of bound C3-fragments on the ECM proteins (**Figures 7A**, **8A**). FHR5 also induced significant FB deposition, whereas FHR1 increased the amount of ECM-bound FB to a lesser extent and at higher serum concentration (**Figures 7B**, **8B**, and data not shown). These results indicate that both FHR1 and FHR5 support alternative pathway activation on ECM proteins.

In the case of FHR5, we further studied this using MaxGel and ECM produced *in vitro* by ARPE-19 cells. These two ECMs of different origin have different composition, laminin dominating in MaxGel and collagen IV in ARPE-derived ECM (**Supplementary Figure 4C**). When these ECMs were exposed to serum in the presence of FHR5, strongly increased deposition of all components of a C3bBbP properdin-stabilized alternative pathway C3 convertase was detected (**Supplementary Figures 4A, B**).

### FHR5 Increases the Deposition of C5b-9 on ECM Proteins

**Figure 9:**
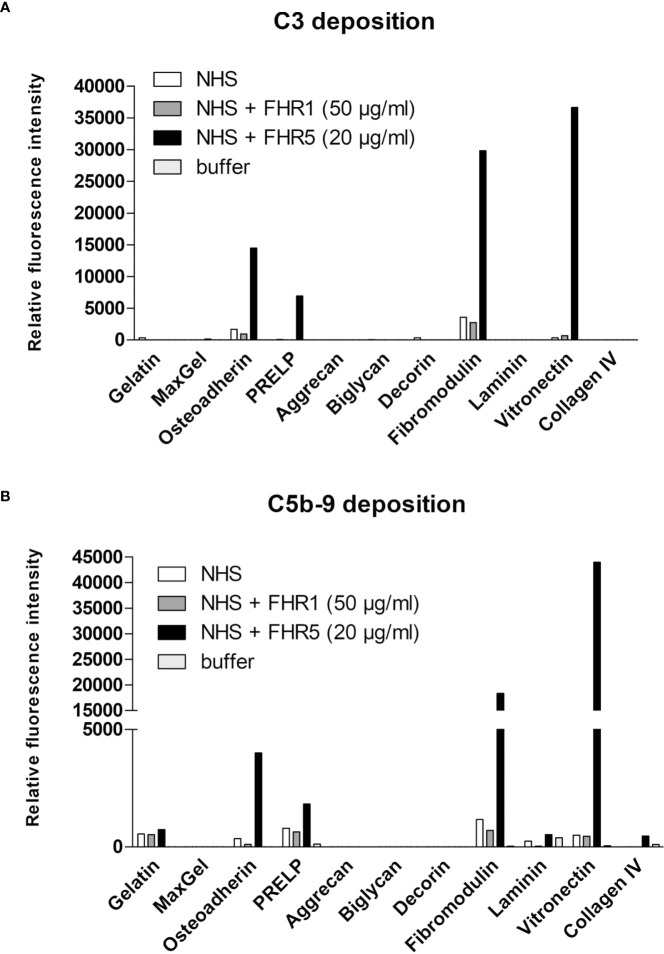
FHR5 increases C5b-9 deposition on ECM ligands. ECM proteins were printed onto nitrocellulose-covered slides and after blocking, proteins were incubated with normal human serum (NHS) in the presence or absence of recombinant FHR1 (50 µg/ml) or FHR5 (20 µg/ml) for 1 hour at 37°C. Bound proteins were detected with polyclonal anti-C3 **(A)** or monoclonal anti-sC5b-9 **(B)** and with the corresponding secondary Ab. Data are representative of two independent experiments.

We used the ECM protein microarray to analyze the effect of FHR1 and FHR5 on terminal complement pathway activation by measuring the deposition of the C5b-9 complex. Immobilized ECM proteins were incubated with NHS with or without the addition of recombinant FHR1 or FHR5. In this assay, FHR5 increased the deposition of C5b-9, along with the deposition of C3 fragments, on osteoadherin, PRELP, fibromodulin and vitronectin (**Figure 9**), while FHR1 had no such effect.

## Discussion

The FH protein family is implicated in the regulation and modulation of complement activation, and members of this protein family were linked to various inflammatory and infectious diseases (46). Whereas the exact functions of the FHR proteins are still to be determined and currently in part controversial, a number of FHR ligands have already been identified. Recent studies show that both FHR1 and FHR5 rather support complement activation in contrast to FH (7, 10). Furthermore, FHR1 and FHR5 were shown to compete with FH for binding to different ligands such as C3b, monomeric/modified C-reactive protein, pentraxin 3, MaxGel (7, 10), and DNA (11), thereby FHRs indirectly enhance complement activation as well.

FHR1 and FHR5 were found in immune deposits in several kidney and eye diseases where excessive complement activation is implicated, indicating that FHRs play an important role in these pathological processes (3, 25, 26, 28, 47). Under these conditions, components of the ECM are exposed and available to interact with complement proteins (31–34). Previously, it was reported that FHR5 binds to MaxGel, an ECM extract (7) and to human laminin (18), an important component of the GBM, and FHR5 was also detected in the ECM of the eye (47); moreover, FHR5 promoted complement activation when bound to MaxGel (7).

In the present study, we show that next to FH, both FHR1 and FHR5 bind to different ECM components relevant in the kidney, eye and joints. Moreover, we show that FHR1 binds to these proteins through its C-terminal domains, while FHR5 binds *via* its middle domains (CCPs 3-7) (**Figures 2**, **3**). The higher signal detected in ELISA for the full-length FHR5 compared with CCPs 3-7 (**Figures 3A, B**) may be due to conformational differences and/or the higher avidity of the dimerized full-length protein and more available epitopes for the detection antibody. These results confirm previous data that FHR5 binds to human laminin by CCPs 5-7 (18). We demonstrate that the middle domains of FHR5 (CCPs 3-7) represent the binding site for immobilized C3b fragment as well, whereas the fluid phase C3b [as it was shown (18)], C3(H_2_O), iC3b and the C3d fragment bind to CCPs 8-9 of FHR5 (**Figure 5**); however, conformational effects and steric hindrances may also influence the observed interactions. FHR1 and FHR5 inhibit the binding of FH to the same ECM components causing reduced cofactor activity of FH (**Figure 4**). These data are in accordance with other studies showing that FHRs compete with FH for different ligands and thereby reduce FH binding and, consequently, its complement activation inhibiting function locally (7, 15). Since the FH splice variant FHL-1 was identified as the predominant complement regulator in Bruch’s membrane (45), its binding to ECM components and the possible modulation of this interaction by FHR1 and FHR5 would be worth studying in the future.

FHR1, FHR4 and FHR5 were shown to directly promote complement activation by binding C3b and allowing formation of the alternative pathway C3 convertase, when bound on surfaces (7, 10, 11, 48, 49). A recent study found direct binding of C3 to FHR1 and proposed that this interaction would allow FHR1 to support complement activation in its vicinity (50). In light of our results with C3(H_2_O) (**Figure 5E**) and the tendency of C3 to tick over at a low rate, we believe it more likely that it is not the abundant C3, but C3(H_2_O) generated by tick-over and/or C3b generated nearby that binds to surface-bound FHRs and serves as a focal point to assemble a C3 convertase, further propagating alternative pathway activation. In our study we demonstrate that both FHR1 and FHR5 increase C3 and FB deposition on the surface of ECM components such as laminin, fibromodulin, osteoadherin and PRELP. Similar to previous results, FHR1 was less effective in triggering complement activation compared to FHR5, and in our assays only the latter enhanced also C5b-9 deposition under the same experimental conditions (**Figures 7**–**9**) (10, 11). To detect FHR1-induced complement activation, higher serum concentrations are required, since the avidity of surface binding depends on initial C3b deposition as well and may also be explained by the bigger dimers of FHR5 compared to the more compact FHR1. In addition, while FHR1 contains only one C3b binding site in its C terminus, in the case of FHR5 surface-bound ligands, including deposited C3b, are recognized by the central domains, while the C terminus remains available for recruiting C3(H_2_O) or C3b (**Figures 3**, **5**).

It was also proposed that FHR5 would promote alternative pathway activation by recruiting properdin *via* the CCPs 1-2 (18, 19). We have re-analyzed this issue using separated physiological properdin forms and found that neither properdin dimers, trimers or tetramers showed significant binding to FHR5 and the other FHRs. In addition, unfractionated properdin that typically contains aggregated properdin multimers did not bind to FHR5 and its fragments, including CCPs 1-2 (**Figure 6**). Thus, while it cannot be excluded that cell-derived properdin may bind to FHR5, serum properdin binding could not be confirmed, in accordance with our previous results (7, 10). However, properdin binding can be detected once a C3 convertase is formed on surface bound FHRs (7, 10, 11).

It is increasingly recognized that the balance between the inhibitor FH and the deregulator FHR proteins determine the extent of complement activation (8, 46), which is influenced by the local and serum levels of FH and the FHRs. Increased FHR to FH ratios were detected in diseases such as IgA nephropathy, AMD, aHUS and rheumatoid arthritis (28, 51–54). Determination of the levels, ligands and functions of the FH family proteins will bring us closer to understand the pathomechanism of these diseases and will likely improve diagnostic, prognostic and therapeutic possibilities.

**Figure 10:**
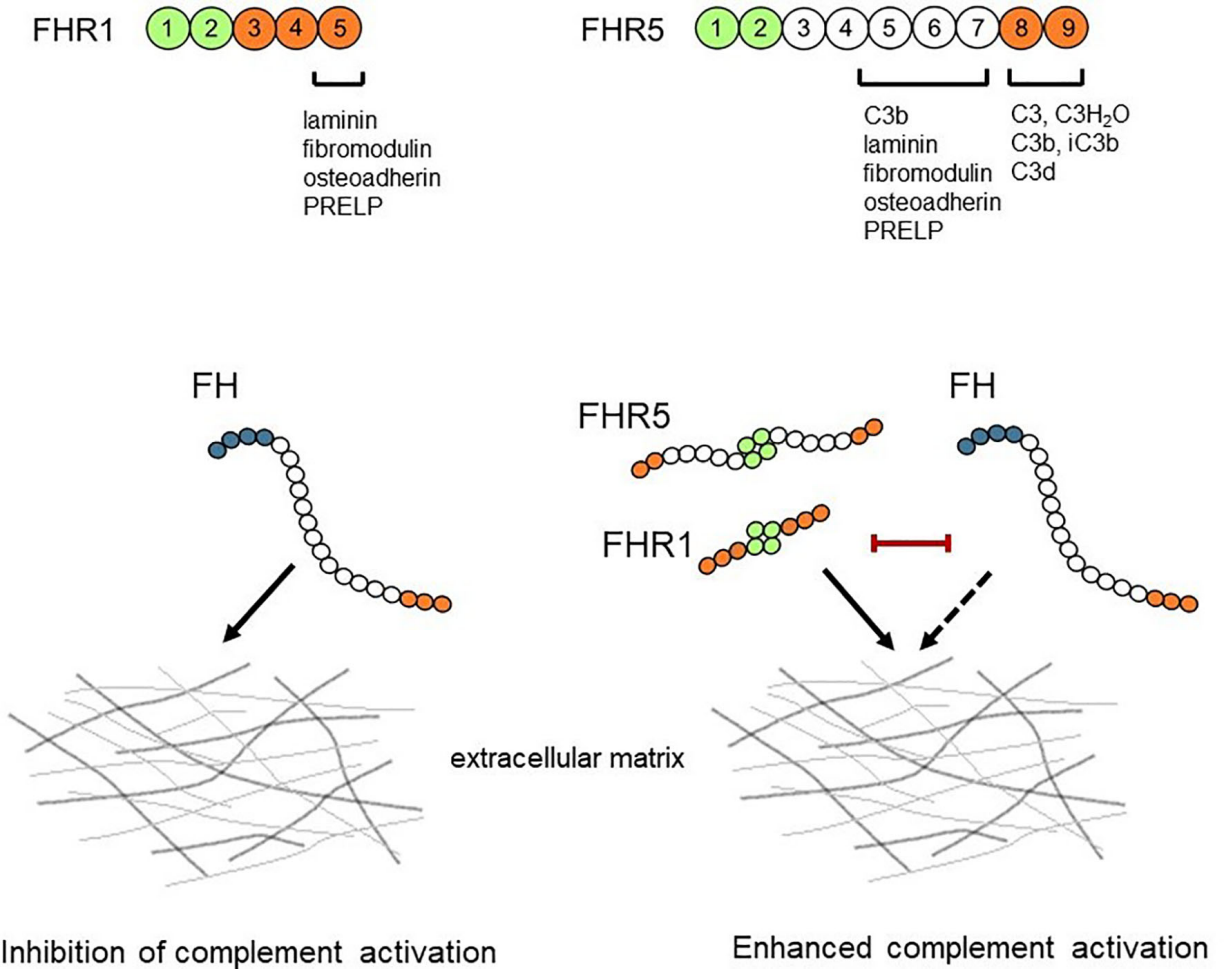
Schematic overview of the interaction between FH/FHRs and the ECM. FHR1 and FHR5 can bind to ECM components and competitively inhibit the binding of FH, thus reducing the complement regulatory activity of FH. The ECM-bound FHR1 and FHR5 may also directly enhance alternative pathway activation by binding C3(H_2_O) and C3b and allowing formation of C3 convertase.

In summary, we show that FHR1 and FHR5 bind to ECM components as does FH; moreover, both FHRs competitively inhibit the binding of FH resulting in reduced complement regulatory activity (**Figure 10**). Furthermore, FHR1 and FHR5 enhance complement activation on ECM proteins indicating that FHRs may contribute to pathological and inflammatory conditions in kidney, eye and joint diseases by modulating the regulatory activity of FH and directly complement activation on ECM.

## Data Availability Statement

The original contributions presented in the study are included in the article/**Supplementary Material**. Further inquiries can be directed to the corresponding author.

## Author Contributions

AP, KP, BU, MC, ÁIC, ZS, and ZB performed experiments. DE, ZP, VPF, and AMB contributed materials. All authors discussed the data. MJ supervised the study. AP and MJ wrote the manuscript with the help of the other authors. All authors listed have made a substantial, direct, and intellectual contribution to the work and approved it for publication.

## Funding

This work was supported in part by the Kidneeds Foundation (Iowa, US), the MedInProt, the National Research, Development and Innovation Fund of Hungary (grants K 109055 and K 125219), the Institutional Excellence Program to ELTE (NKFIH-1157/8/2019, D11206), the Hungarian Academy of Sciences (0106307), the European Union’s Horizon 2020 research and innovation programme under grant agreement No. 899163 (SciFiMed), all to MJ; the National Institutes of Health (grant R01HL112937 to VPF), and the Swedish Research Council (2018-02392, to AMB).

## Author Disclaimer

Parts of this work were presented at the 16^th^ European Meeting on Complement in Human Disease, September 8-12, 2017, Copenhagen, Denmark (*Mol. Immunol.* 2017, 89:145), and at the 27^th^ International Complement Workshop, September 16-20, 2018, Santa Fe, New Mexico, USA (*Mol. Immunol.* 2018, 102:196).

## Conflict of Interest

The authors declare that the research was conducted in the absence of any commercial or financial relationships that could be construed as a potential conflict of interest.

## Publisher’s Note

All claims expressed in this article are solely those of the authors and do not necessarily represent those of their affiliated organizations, or those of the publisher, the editors and the reviewers. Any product that may be evaluated in this article, or claim that may be made by its manufacturer, is not guaranteed or endorsed by the publisher.

## Abbreviations

aHUS, atypical hemolytic uremic syndrome; AMD, age-related macular degeneration; BM, Bruch’s membrane; BSA, bovine serum albumin; C3G, C3 glomerulopathy; CCP, complement control protein domain; DPBS, Dulbecco’s phosphate-buffered saline; ECM, extracellular matrix; FB, factor B; FD, factor D; FH, factor H; FHR, factor H-related; FHR1, factor H-related protein 1; FHR4, factor H-related protein 4; FHR5, factor H-related protein 5; FI, factor I; FP, factor P; GBM, glomerular basement membrane; HSA, human serum albumin; NHS, normal human serum; PRELP, proline/arginine-rich end leucine-rich repeat protein.
