# Supplementary material for: Complement Factor H-Related Proteins FHR1 and FHR5 Interact With Extracellular Matrix Ligands, Reduce Factor H Regulatory Activity and Enhance Complement Activation
Source: Front Immunol. 2022 Mar 22;13:845953. doi: 10.3389/fimmu.2022.845953 (PMC8980529; doi:10.3389/fimmu.2022.845953)
Supplement: Supplementary file 1 [file DataSheet_1.pdf]

Supplementary Material

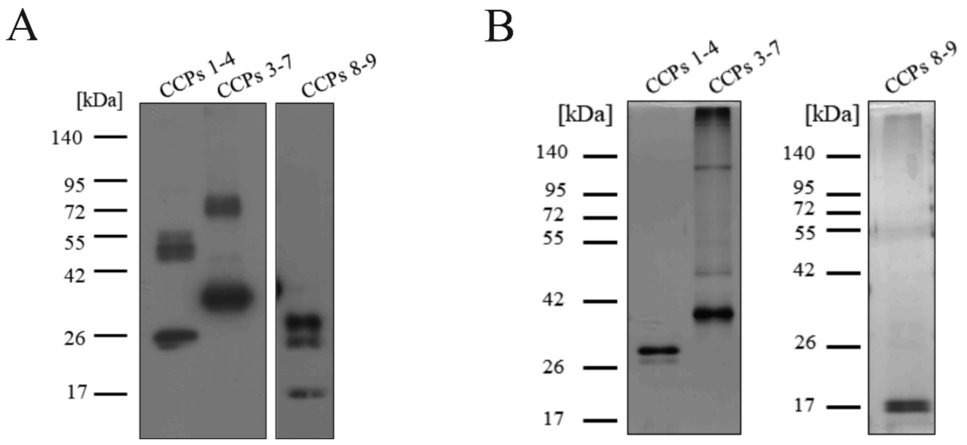

Supplementary Figure 1. The His-tagged FHR5 fragments comprising CCPs 1-4, CCPs 3-7 and CCPs 8-9 were cloned using pBSV-8His Baculovirus expression system, expressed in *Spodoptera frugiperda* (Sf9) insect cells, and purified by nickel affinity chromatography. (A) Proteins were separated by 10 % SDS-PAGE and Western blotting. The blot was developed using polyclonal goat anti-human FHR5 Ab. The upper bands represent dimers of the proteins. (B) The purity of the proteins (1 µg/lane) was analyzed by silver staining.

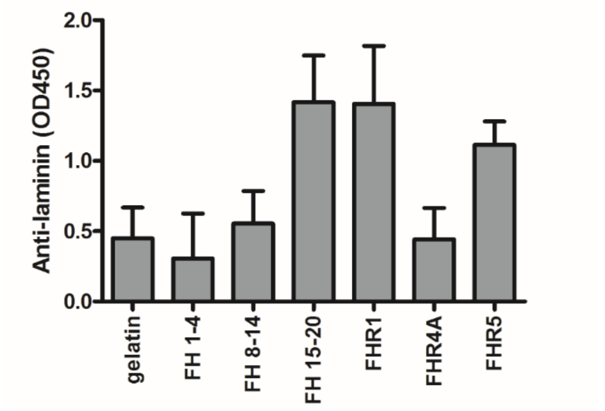

Supplementary Figure 2. Laminin binding to immobilized FH fragments and FHRs in ELISA. Equimolar amounts of gelatin, the recombinant FH fragments comprising CCPs 1-4, 8-14 and 15-20, and the recombinant FHR proteins FHR1, FHR4A and FHR5 were immobilized in microplate wells. After incubation with human laminin, laminin binding was detected with anti-laminin antibody. Data show means + SD from three experiments.

A

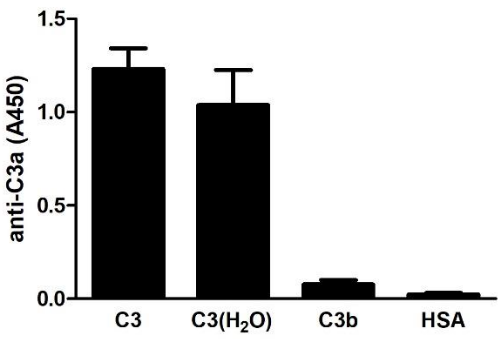

B

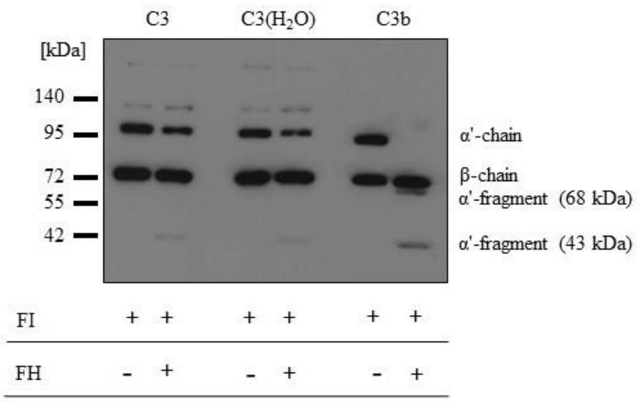

Supplementary Figure 3. Analysis of $C3(H_2O)$ by ELISA and cofactor assay. (A) ELISA was performed to detect the C3a part in intact C3 and $C3(H_2O)$. To this end, 5 µg/ml C3, $C3(H_2O)$, C3b and HSA were immobilized and, after blocking, rabbit anti-C3a antibody was added, followed by HRP-conjugated swine anti-rabbit Ab. (B) 140 nM C3, $C3(H_2O)$ and C3b were incubated with 300 nM factor I in the presence (+) or absence (-) of FH (320 nM), as indicated, for 1 hour at 37°C. Samples were loaded onto 7% polyacrylamide gel and subjected to SDS-PAGE and Western blotting. C3 fragments were detected using a HRP-conjugated anti-human C3 Ab and ECL detection kit. A representative blot is shown.

A

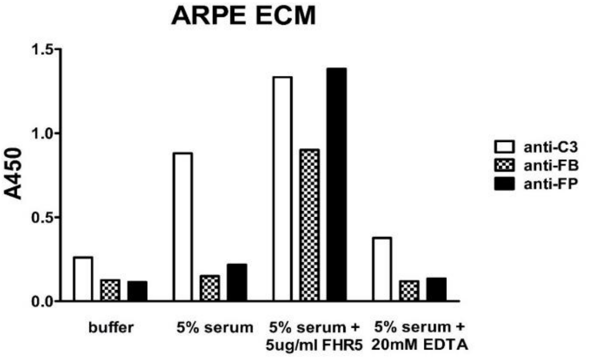

B

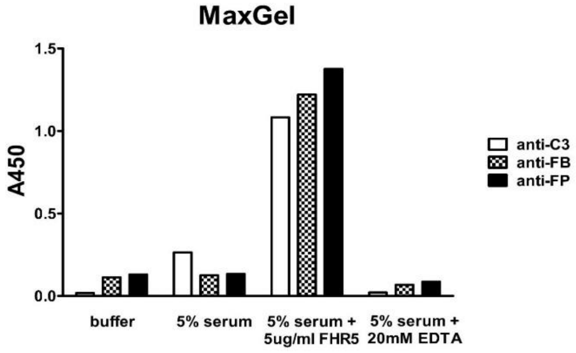

C

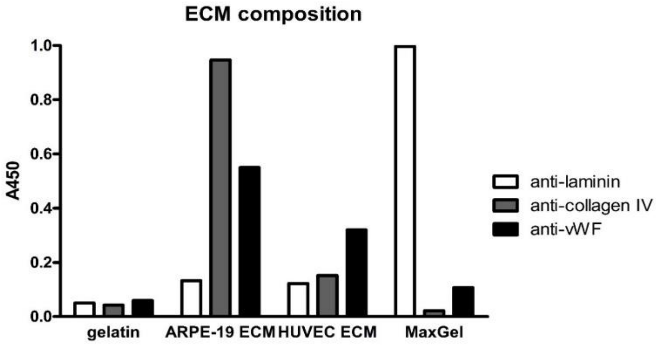

Supplementary Figure 4. FHR5 enhances complement activation when bound to ARPE-derived ECM and MaxGel. (A) Adult retinal pigment epithelial cells (ARPE-19) were seeded onto 96-well cell culture plate coated with 0.2% gelatin and cultured until confluence. Cells were detached with 20 mM EDTA in PBS at 37ºC. (B) MaxGel diluted 1:30 was immobilized on ELISA plate. After blocking, 5% normal human serum (NHS) was added, as indicated, for 30 min at 37ºC. Bound proteins were detected with anti-human C3, anti-human factor B (FB) and anti-properdin (FP) Abs. (C) ARPE-19 and HUVEC cells were cultured and the cell-free ECM was analyzed in comparison with MaxGel for the presence of ECM components, which were detected with antibodies specific for laminin, collagen IV or von Willebrand factor (vWF). Representative results from two experiments are shown.
